# Effects of Gabapentinoids Premedication on Shoulder Pain and Rehabilitation Quality after Laparoscopic Cholecystectomy: Pregabalin versus Gabapentin

**DOI:** 10.1155/2018/9834059

**Published:** 2018-07-09

**Authors:** Mohamed Said Nakhli, Mohamed Kahloul, Chawki Jebali, Walid Frigui, Walid Naija

**Affiliations:** ^1^Department of Anesthesia and Intensive Care, Sahloul Academic Hospital, Sousse, Tunisia; ^2^University of Medicine “Ibn Al Jazzar”, Sousse, Tunisia; ^3^Emergency Medical Service, Sahloul Academic Hospital, Sousse, Tunisia

## Abstract

**Introduction:**

Gabapentinoids are increasingly used in preoperative premedication despite controversial results. The aim of our study was to evaluate the effects of preemptive use of gabapentin or pregabalin on postoperative shoulder pain and rehabilitation quality after laparoscopic cholecystectomy.

**Methods:**

This is a clinical trial comparing the effects of a preoperative premedication with 600 mg of gabapentin or 150 mg of pregabalin versus placebo on postoperative pain and recovery quality after laparoscopic cholecystectomy. Premedication was taken 2 hours before the surgery beginning. Ninety patients were included and randomized into 3 groups (gabapentin, pregabalin, and placebo). The anesthetic protocol was the same for all patients. Primary endpoint was the shoulder pain intensity at the 48th postoperative hour. Secondary endpoints were postoperative nausea and vomiting (PONV), sleep quality during the first night, and the onset time for the first standing position.

**Results:**

During the first 48 postoperative hours, the gabapentin and pregabalin groups had significantly lower shoulder pain than the placebo group (*p* < 0.05). In gabapentinoids groups, the incidence of PONV was lower and the sleep quality during the first postoperative night was better with significant results. Mean Spiegel scores were 22.43 ± 1.45, 22.30 ± 1.44, and 17.17 ± 1.66, respectively, in pregabalin, gabapentin, and placebo groups (*p* < 0.05). The delay for the first standing position was 14.9 ± 4.9 hours in the pregabalin group, 9.7 ± 3.6 hours in the gabapentin group, and 21.6 ± 2.1 hours in the placebo group. No superiority was found between gabapentin and pregabalin.

**Conclusion:**

Preemptive premedication with gabapentinoids can enhance postoperative rehabilitation quality after laparoscopic cholecystectomy by reducing postoperative shoulder pain, decreasing PONV incidence, and improving sleep quality during the first postoperative night. This trial is registered with ClinicalTrial.gov (NCT03241875).

## 1. Introduction

Laparoscopic cholecystectomy is one of the most widely performed surgeries in the world [1]. Its postoperative follow-up is usually simple allowing ambulatory practices [1, 2]. However, postoperative pain, especially scapular ones, seems to be a hindrance to a quick postoperative recovery [3]. In fact, postoperative shoulder pain is a common side effect after laparoscopic procedures. It seems to have a multifactorial mechanism, but the most reported theory is carbon dioxide gas persistence between the right diaphragm and the hepatic dome. It appears mainly after patient verticalization and it may remain even after standard analgesic medications. In addition, the high emetic potential of laparoscopic procedures with an incidence exceeding 75% when no specific prophylaxis is prescribed can extend the length of hospital stay [4].

Gabapentin and pregabalin are two drugs belonging to the gabapentinoids family. They are classically known as antiepileptic treatments. However, they have been used in other conditions such as neuropathic, diabetic, and postherpetic pain [5]. They have few drug interactions and side effects with close pharmacokinetic profiles justifying their interest in the perioperative context [5, 6].

Preemptive administration of gabapentinoids can reduce postoperative pain intensity even after laparoscopic surgery [7]. It can also alleviate preoperative anxiety and stress response to surgical stimuli and decreases anesthetic consumption and postoperative nausea and vomiting (PONV) [6, 8–10].

The aim of this study was to evaluate the effects of preemptive use of gabapentin or pregabalin on postoperative shoulder pain and recovery parameters after laparoscopic cholecystectomy.

## 2. Methods

After obtaining institutional ethic committee approval and patients' informed consent, this randomized, double-blind clinical trial was conducted jointly between the anesthesia and the visceral surgery departments of Sahloul Teaching Hospital (Sousse, Tunisia). This study was conducted between December 2016 and August 2017. This trial was registered with ClinicalTrial.gov (NCT03241875).

Patients aged 18–65 years, with American Society of Anesthesiologists (ASA) physical status 1 and 2, and scheduled for laparoscopic cholecystectomy under general anesthesia were enrolled in this study. Exclusion criteria were as follows: allergy to gabapentin or pregabalin, history of psychiatric disorders, chronic neuroleptic treatment, history of renal and/or hepatic disorders, pregnant and/or breastfeeding women, complicated lithiasis pathology, conversion to laparotomy, and perioperative complications (surgical difficulties, difficult intubation, bronchospasm, anaphylaxis, etc).

Fixing the *α* risk at 0.05 and the study power at 80% and hoping a 30% decrease in shoulder pain intensity at the 48th hour postoperatively, the minimum sample size was estimated at 29 patients per group. Sample calculation was made by the software Epi Info 6.0.

Patients were randomized into 3 groups of 30 each. Patients of group 1 received 150 mg of pregabalin (2 capsules of 75 mg), those of group 2 received 600 mg of gabapentin (2 capsules of 300 mg), and those of group 3 received 2 capsules of placebo. Capsules were arranged in nontransparent and numbered envelopes. They were given to patients during preoperative visit to be taken 2 hours before arrival in the operating room. No other premedication was prescribed.

In the operating room, all patients had standard monitoring (electrocardiogram, noninvasive blood pressure, pulse oximetry, capnography, anesthetic gas analyzer, and neuromuscular monitoring). Anesthesia induction was carried out by propofol at the dose of 3 mg/kg, remifentanil at the dose of 1 *µ*g/kg over one minute, and cisatracurium at the dose of 0.15 mg/kg. Anesthesia was maintained by isoflurane in a mixture of 50% oxygen and 50% air. Remifentanil doses and expired isoflurane concentrations were adjusted intraoperatively to maintain a hemodynamic profile (arterial pressure and heart rate) not exceeding ±20% from baseline. Cisatracurium was reinjected at the dose of 0.03 mg/kg depending on neuromuscular blockade monitoring. PONV risk was assessed by the Apfel score (a four-criteria score used to estimate the probability of experiencing PONV) and prevented by dexamethasone 8 mg and ondansetron 4 mg. Normal saline was used for intraoperative vascular loading at the dose of 4 ml/kg/h. Tidal volume was fixed at 6 ml/kg of ideal weight, and respiratory rate was adjusted to maintain EtCO2 between 30 and 35 mmHg.

Postoperative analgesia was anticipated 30 minutes before the end of surgery by 1 g of paracetamol and 20 mg of nefopam. All patients were extubated in the operating room after complete decurarization. They were monitored in the post-anesthesia care unit for one hour and then transferred to the visceral surgery ward. Postoperative analgesia was performed with 1 g of paracetamol and 20 mg of nefopam given four times a day. Postoperative vomiting was managed by 4 mg of ondansetron.

Primary endpoint was shoulder pain intensity at the 48th postoperative hour assessed by the Visual Analog Scale (VAS) ranging from 0 to 10. Secondary endpoints were PONV incidence, sleeping quality assessed by Spiegel Scale (a very simple and widely used scale with six questions about sleep; total score below 15 signifies a pathological sleep and a score above 20 signifies a good sleep), and time for the first standing position.

Statistical analysis was performed using SPSS software version 20.0 for windows (IBM Corp., USA). Categorical variables were expressed in percentages. Normal distribution of continuous variables was tested using the Kolmogorov–Smirnov test. They were expressed as means or medians according to their distribution.

Categorical variables were analyzed using chi-squared test. Student's *t*-test was used to compare means of normally distributed variables, whereas the Mann–Whitney test was used for non-normally distributed ones. A value of *p* < 0.05 was considered to be statistically significant.

## 3. Results

This study enrolled 90 patients undergoing scheduled laparoscopic cholecystectomy. All of them were followed up until the end of the study. Demographic characteristics were comparable between all groups. Insufflation pressure and duration of surgery, anesthesia, and pneumoperitoneum were also comparable between the 3 groups (Table 1).

**Figure 1:**
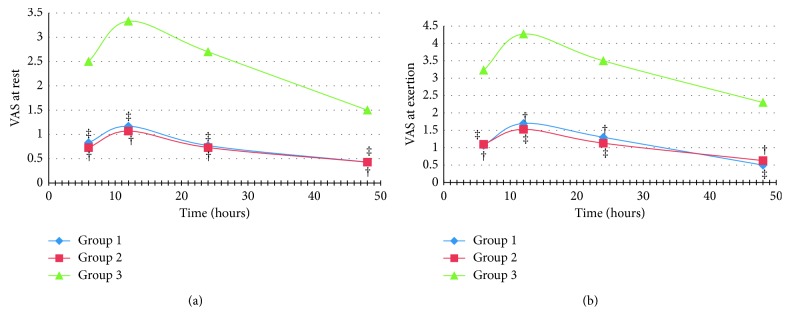
Postoperative shoulder pain intensity ((a) at rest and (b) at exertion). ^†^*p* < 0.05 between groups 1 and 3. ^‡^*p* < 0.05 between groups 2 and 3.

At the 48th hour after surgery, the overall incidence of postoperative shoulder pain was 51.1%. It was 13.3% in the pregabalin group, 12.2% in the gabapentin group, and 25.6% in the control group (*p*=0.003). Postoperative shoulder pain occurred after the 6th postoperative hour in all cases. Its intensity at rest and at exertion was significantly lower in the gabapentinoids groups than in the control one. When comparing the pregabalin group to the gabapentin one according to postoperative shoulder pain intensity, no significant difference was found (Figure 1).

PONV incidence was significantly higher in the control group than in the gabapentinoids ones; however, they were at the same risk of PONV as assessed by the Apfel score. No difference was found when comparing the pregabalin group to the gabapentin group (Table 2).

During the first postoperative night, sleeping quality was significantly better in gabapentinoids groups (*p* < 0.05). Average Spiegel scales were 22.43 ± 1.45 in the pregabalin group, 22.30 ± 1.44 in the gabapentin group, and 17.17 ±1.66 in the control group. Sleeping quality was comparable between the pregabalin group and gabapentin group (Table 2).

Postoperative deambulation was significantly earlier in the gabapentinoids groups. No significant difference was found between the pregabalin and gabapentin groups (14.9 ± 4.9 hours in the pregabalin group, 9.7 ± 3.6 hours in the gabapentin group, and 21.6 ± 2.1 hours in the control group; *p* < 0.05) (Table 2).

Dizziness was the unique type of side effects documented in the gabapentinoids groups (9 patients in the gabapentin group versus 11 patients in the pregabalin group). This adverse effect was reported only before anesthesia induction.

## 4. Discussion

Our results suggest that a premedication with a gabapentinoid (150 mg of pregabalin or 600 mg of gabapentin) can reduce the intensity of postoperative shoulder pain after laparoscopic cholecystectomy. It can also reduce the incidence of PONV, shorten the time to the first standing position, and improve sleep quality during the first postoperative night. However, no superiority was found when comparing the two gabapentinoids.

Gabapentin and pregabalin are known as antiepileptic drugs. However, they have other reported uses due to their analgesic and anxiolytic effects [5, 10, 11]. They have comparable pharmacokinetic characteristics. The half-life of elimination is around 6 to 8 hours. The bioavailability is about 35 to 60% for gabapentin and 90% for pregabalin. Peak plasma concentration is reached in less than 2 hours for gabapentin versus 30 to 120 min for pregabalin [9, 12].

Several studies have shown the effect of gabapentinoids use as anesthetic adjuvant in the reduction of postoperative pain and analgesic requirements [7, 9, 10, 13–17]. Main studied surgeries were laparoscopic cholecystectomy, hysterectomy, mastectomy, and tonsillectomy. A low dose ranging from 600 to 1200 mg of gabapentin and from 150 to 300 mg of pregabalin was used in these trials without any reported significant adverse effect [18–20].

Although a single preemptive administration was the most prescribed modality, other studies kept the treatment for 2 to 5 days postoperatively. The meta-analysis of Lam et al. including 74 protocols of pregabalin use in different surgeries did not found any significant difference in postoperative pain reduction when comparing a single preemptive administration to an extended postoperative administration. When comparing different gabapentinoids drugs especially for laparoscopic cholecystectomy, findings are in favour of pregabalin [21].

Mishra et al. compared 150 mg of pregabalin to 900 mg of gabapentin in postoperative pain after laparoscopic cholecystectomy. Pain intensity and analgesic requirements were significantly lower in the pregabalin group at the expense of an increase in postoperative sedation [15]. Similar results were found by Eidy et al. by their clinical trial showing a significant decrease in postoperative pain and pethidine requirements in the pregabalin group compared to the gabapentin group [13].

Although analgesic effects of gabapentinoids are well described in postoperative pain, few data are available about the postoperative shoulder pain which is particularly frequent in post-laparoscopic surgery. It has a mechanical mechanism, as it is related to residual gas in the peritoneal cavity and it occurs mainly after patients' mobilization. Valadan et al. conducted a clinical trial enrolling 40 patients scheduled for laparoscopic ovarian surgery. After premedication with 600 mg of gabapentin, a significant decrease in the intensity of postoperative shoulder pain at rest and at exertion was noted up to the 12th hour postoperatively [8]. This analgesic effect was found with smaller dose of gabapentin (150 mg) in the study of Nutthachote et al. enrolling patients scheduled for laparoscopic gynecologic surgeries [22]. However, other studies showed different results [23]. For instance, the postoperative shoulder pain reduction due to preemptive premedication by gabapentinoids started at the 6th postoperative hour and reached more than 70% at the 12th postoperative hour. The same results were found for pain intensity following trocar insertion. In a systematic review analyzing 52 trials and enrolling 1774 patients, Tiippana et al. had evaluated the effect of preemptive gabapentin and pregabalin administration in postoperative pain after various surgical procedures. The postoperative pain intensity reduction started at the second postoperative hour [6].

Analgesic effects of gabapentinoids are due to their anxiolytic, antinociceptive, and opioid-like characteristics which are mainly explained by a central neural sensitization. Gabapentinoids have also an antihyperalgesic effect by their interaction with voltage-gated calcium channels of the spinal cord and the dorsal root ganglia [24]. Finally, a probable modulation of glutamate receptors by gabapentinoids has been reported [25].

By reducing postoperative pain, gabapentinoids seems to be interesting in inducing a quick postoperative rehabilitation allowing ambulatory practices after laparoscopic cholecystectomy.

However, the high incidence of nausea and vomiting after laparoscopic procedures must be considered before patient discharge as it compromises the oral intake. Various pharmacological treatments are usually used in the operating room for antiemetic purpose. Dexamethasone–ondansetron association is the most prescribed medication. Recently, several studies have evaluated the antiemetic effects of gabapentinoids with controversial results.

In a quantitative analysis of evidence from randomized controlled clinical trials, Achuthan et al. evaluated gabapentin prophylaxis for PONV in abdominal surgeries. Seventeen studies enrolling 1605 patients were analyzed. The incidence of nausea was reduced by 24% and the incidence of vomiting was reduced by 38% in the gabapentin group [26]. Ho et al. found similar results in a systematic review concluding to a lower incidence of PONV due to a preemptive gabapentin administration (RR 0.58; CI 95% 0.39–0.86) [19]. Gabapentinoids antiemetic properties are not well described. Several hypotheses have been reported such as a reduction of opioid requirements during and after anesthesia, a reduction of anesthetic gas consumption, and a reduction of tachykinin activity [27].

Finally, given analgesic and antiemetic properties of gabapentinoids, together with their sedative characteristics, an improvement in patients sleep quality especially during the first postoperative night was expected and evaluated in our study.

Lunn et al. evaluated analgesic and sedative effects of perioperative gabapentin in a double-blind trial enrolling patients scheduled for total knee replacement. Assessed by the Nonrestorative Sleep Scale (NRSS), the quality of sleep was better in the gabapentin group during the first two postoperative nights [28].

However, Eloy et al. found different results when they studied the effect of gabapentin on postoperative pain and sleep quality in patients undergoing total knee arthroplasty, total hip arthroplasty, or a hip fracture repair. Their findings indicate that gabapentin does not improve sleep quality assessed by the Pittsburg Sleep Quality Index. The small dose of gabapentin and the small study sample must be considered in results interpretation [29]. Assessed by Spiegel score, sleep quality in our study was better in gabapentinoids groups without significant difference between gabapentin and pregabalin.

Preemptive premedication with 600 mg of gabapentin or 150 mg of pregabalin improves several parameters of postoperative rehabilitation after laparoscopic cholecystectomy. It reduces significantly the intensity of postoperative shoulder pain, decreases the incidence of PONV, improves the quality of sleep during the first night, and shortens the time to first standing position. However, some limitations should be considered such as the nonevaluation of analgesic requirements during the postoperative period and the absence of the pharmacoeconomic component in our study.

## 5. Conclusion

Preemptive premedication with gabapentin or pregabalin before laparoscopic cholecystectomy can be interesting in postoperative rehabilitation allowing ambulatory practices. In fact, it can reduce postoperative shoulder pain and PONV incidence. It can also improve the sleep quality during the first postoperative night. However, the optimal doses of gabapentinoids and the duration of treatment are not yet well established.

## Figures and Tables

**Table 1:** Sociodemographic and intraoperative characteristics.

	Group 1 *n*=30	Group 2 *n*=30	Group 3 *n*=30	*p*
Age (years)	42.7 ± 13.7	47.1 ± 13.1	43.5 ± 13.6	0.433
BMI (kg/m^2^)	26.3 ± 2.4	28.1 ± 4.5	25.6 ± 3.3	0.081
Gender *n* (%)				
Male	7 (23.3)	6 (20)	6 (20)	0.93
Female	23 (76.7)	24 (80)	24 (80)	
Apfel score: *n* (%)				
1	4 (13.3)	6 (20)	4 (13.3)	0.61
2	26 (86.7)	24 (80)	25 (83.3)	
3	0 (0)	0 (0)	1 (3.3)	
ASA status *n* (%)				
1	26 (86.6)	23 (76.6)	25 (83.3)	0.58
2	4 (13.4)	7 (23.4)	5 (16.7)	
Surgery duration (min)	51 ± 14.4	56.2 ± 17.7	50.7 ± 15.9	0.398
Pneumoperitoneum duration (min)	37.6 ± 15.2	37.3 ± 16.3	37.1 ± 13.6	0.983
Insufflation pressure (mmHg)	11.6 ± 0.6	11.6 ± 0.7	11.5 ± 0.7	0.72
Anesthesia duration (min)	63.1 ± 16	68 ± 18.4	63.9 ± 15.5	0.47

**Table 2:** Effects of gabapentinoids on PONV, sleep quality, and first standing position.

	Group 1 *n*=30	Group 2 *n*=30	Group 3 *n*=30
Postoperative nausea *n* (%)			
2nd hour	4 (13.3)^†^	8 (26.7)^‡^	25 (83.3)
6th hour	5 (16.7)^†^	2 (6.7)^‡^	22 (73.3)
12th hour	0 (0)^†^	3 (10)^‡^	12 (40)
Postoperative vomiting *n* (%)			
2nd hour	5 (16.7)^†^	4 (13.3)^‡^	21 (70)
6th hour	1 (3.3)^†^	4 (13.3)^‡^	12 (40)
12th hour	0 (0)^†^	0 (0)^‡^	4 (13.3)
Spiegel score (mean)	22.43 ± 1.45^†^	22.30 ± 1.44^‡^	17.17 ± 1.66
Time for the first standing position (hr)	14.9 ± 4.9^†^	9.7 ± 3.6^‡^	21.6 ± 2.1

^†^ *p* < 0.05 between groups 1 and 3. ^‡^*p* < 0.05 between groups 2 and 3.

## Data Availability

Readers can access the data underlying the findings of our study, by sending an e-mail to the corresponding author.
